# Redefining professionalism to improve health equity in competency based medical education (CBME): A qualitative study

**DOI:** 10.12688/mep.20489.1

**Published:** 2024-10-18

**Authors:** Linda Bakunda, Rachel Crooks, Nicole Johnson, Kannin Osei-Tutu, Aleem Bharwani, Emmanuel Gye, Daniel Okoro, Heather Hinz, Shelley Nearing, Leah Peer, Aliya Kassam, Penelope Smyth, Pamela Chu, Shannon Ruzycki, Mala Joneja, Doreen Rabi, Cheryl Barnabe, Pamela Roach

**Affiliations:** 1 Department of Community Health Sciences, University of Calgary, Calgary, Alberta, Canada; 2 Department of Family Medicine, University of Calgary, Calgary, Alberta, Canada; 3 Department of Pediatrics, University of Calgary, Calgary, Alberta, Canada; 4 Department of Medicine, University of Calgary, Calgary, Alberta, Canada; 5 Department of Psychiatry, University of Calgary, Calgary, Alberta, Canada; 6 Department of Medicine, University of Alberta, Edmonton, Alberta, Canada; 7 Department of Obstetrics and Gynecology, University of Calgary, Calgary, Alberta, Canada; 8 Division of Rheumatology, Queen's University, Kingston, Ontario, Canada

**Keywords:** Competency-based medical education, anti-racism, professionalism

## Abstract

**Purpose:**

There is a pressing need to address all forms of anti-oppression in medicine, given systemic harm and inequities in care and outcomes for patients and health care professionals from equity-deserving groups. Revising definitions of professionalism used in competency-based education can incorporate new professional competencies for physicians to identify and eliminate the root causes of these inequities. This study redefined the CanMEDS *Professionalism* definition to centre perspectives of equity-deserving groups.

**Methods:**

In this qualitative study there were two phases. The authors conducted individual semi-structured interviews with participants representing equity-deserving population groups to understand their perspectives on and iteratively build a definition of medical professionalism. Then, the authors undertook a consensus-building process, a modified nominal group technique, using focus groups with community members from equity-deserving groups and healthcare providers to verify findings and arrive at an updated definition of medical professionalism.

**Results:**

Four main themes were identified: 1) healthcare at the margins; 2) equity-oriented domains of professionalism; 3) structural professionalism; and 4) supporting improved professionalism. These themes were incorporated into a consensus-based definition of medical professionalism, with a focus on anti-oppression, anti-racism, accountability, safety, and equity.

**Conclusions:**

The authors propose a new definition of medical professionalism that embeds anti-oppression, including anti-racism, as critical competencies in clinical practice and education.

## Introduction

Given the evidence of structural, systemic, and interpersonal discrimination in medicine ^1^ and broader society, addressing all forms of inequity and explicitly supporting anti-racist ^2^ action must be a medical education priority. A collaborative, inclusive approach to re-examine and re-define medical professionalism as outlined by CanMEDS ^3, 4^ is required to encourage anti-oppressive education and practice ^5^. The CanMEDS competency framework, widely used beyond its Canadian foundations, outlines seven roles expected of physicians. The Royal College of Physicians and Surgeons of Canada (RCPSC) has started the CanMEDS 2025 project, aiming to update their CanMEDS 2015 Physician Competency Framework ^6^ and aligning with the framework's regular update intervals ^7^. RCPSC state that the update intends to strengthen anti-racism and anti-oppression efforts, champion equity, diversity, inclusion, and accessibility goals, and proactively address societal needs, including those outlined by the Truth and Reconciliation Commission ^8^. All CanMEDS competencies are undergoing revision, and redefining Professionalism competency allows for an opportunity to address both structural aspects and individual behaviors. Both the CanMEDS framework and Accreditation Council for Graduate Medical Education (ACGME) currently describe the “ *Professional* role” and “ *Professionalism*” respectively, with defined competencies and developmental milestones toward professionalism expertise ^9, 10^. The CanMEDS *Professional* role includes a commitment to the patient, society, profession, and the physician themselves. The shift towards competency-based medical education (CBME) ^11, 12^ reflects the professional role requirement of self-regulation by defining measurable and evaluable outcomes.

The current *Professional* role was developed through an iterative process that included physicians with the RCPSC and partnering organizations. While data on the diversity and representation of people from marginalized identities involved in creating the historical definition of professionalism is not available, there are reasons to suggest that the representation was inadequate. For example, there are few (or no) Black, Indigenous, People of Colour, women, gender diverse or transgender, and disabled or other ability physician and health care leaders in Canada ^13– 15^. A lack of diverse representation in the group defining professionalism may very likely exclude domains of professionalism that are critical to equity-deserving groups ^16^. Incorporating individuals from various backgrounds, encompassing gender, cultures, ethnicities, and religions brings unique experiential knowledge to discussions that aid in promoting equality ^17^. While the current *Professional* role calls for “respect of diversity” ^18^, evidence demonstrates that this call has been ineffective at promoting equitable care and educational experiences ^1, 19^. A definition of professionalism as part of professional competence that includes an explicit commitment to anti-oppression broadly will create an obvious expectation for medical learners and physicians to oppose discrimination in all forms and to explicitly promote racial equity and justice. The purpose of this study was to take an ethically and culturally safe approach to develop an updated definition of professionalism that incorporates anti-racism and anti-oppression and that can be used to expand existing competency training objectives.

## Methods

**Figure 1.**
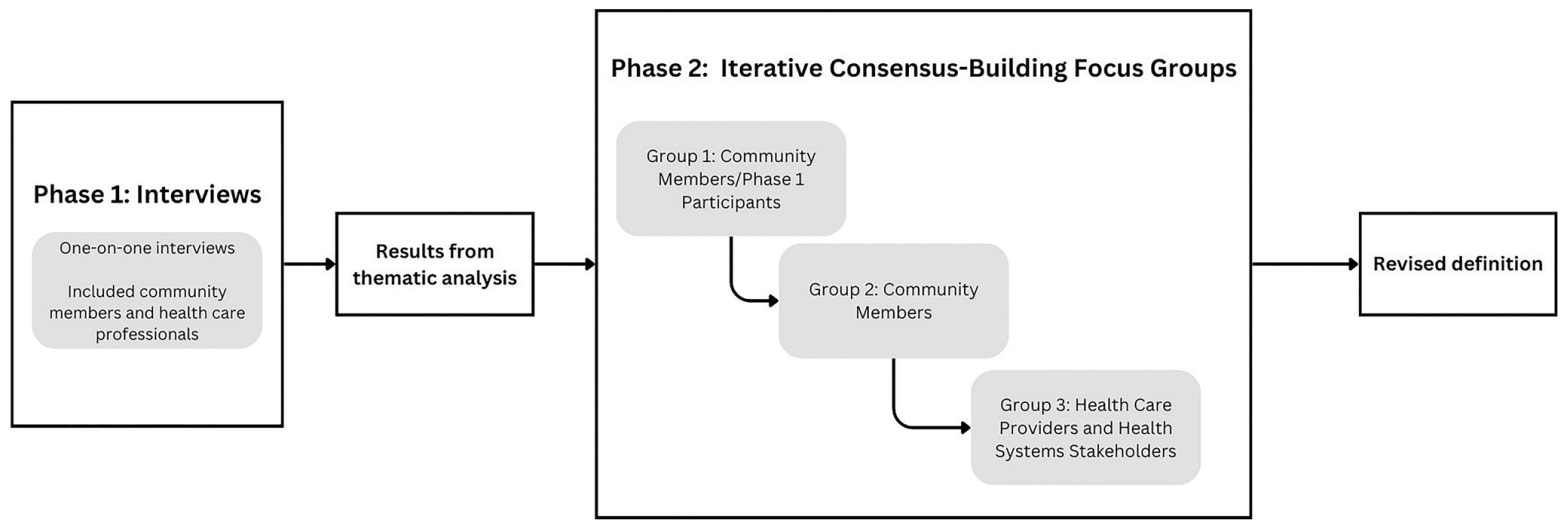
Overview of the study phases and design.

Special attention was required while designing this qualitative study to ensure there was representation of experiences from equity-deserving groups in medicine and the academy and that we grounded ourselves in the concepts of ethical space ^20^. While urgent attention is needed to specifically address anti-oppression and anti-racism in the professionalism expectations in academic medicine, the impact of the intersectionality ^21^ of race and other identities on health must not be ignored so they that do not inadvertently perpetuate oppressive structures for select groups. Therefore, we addressed intersectionality and included in our definition of underrepresented groups members of Black, Indigenous, and People of Colour (BIPOC) communities, people belonging to minority religious groups, LGBTQ2S+ community members, and people living with diverse visible or invisible disabilities. Participants were invited to self-identify with group(s) that they felt represented them, rather than having researchers assign rigid categories of identity. The authors also acknowledge that individuals within the BIPOC group have distinct experiences and BIPOC is not a term used to homogenize a large group of diverse people, but the group is reported throughout the results as an aggregate to avoid risk of identification of individual participants. The study took place in two phases ( Figure 1). To ensure rigorous data collection and analysis, member checking and peer debriefing were used throughout the process ^22^. Member checking is a technique used to enhance trustworthiness and improve credibility, where researchers return findings to participants to verify the accuracy and ensure findings resonant with their experiences. Peer debriefing is used to establish trustworthiness and credibility of the findings. A researcher and an impartial team member engaged in discussions about the study’s findings and asked questions to help the researcher recognize how their personal perspectives and values might influence the results. This questioning minimizes bias in the analysis ^22^. The project adheres to the Declaration of Helsinki on ethical research involving human subjects ^23^. This project was approved by the University of Calgary Conjoined Health Research Ethics Board (REB21-0036) in April 2021.

### Phase One Methods: Understanding professionalism and health equity

In phase one of the study, participants with diverse identities were recruited using purposive and snowball sampling via social media, pre-existing community engagement teams, and relevant community organizations to participate in qualitative semi-structured one-on-one interviews. Interviews were conducted via Zoom web conferencing software ^24^ by two research assistants with experience in qualitative health research (LB and RC) between March 2022 and May 2022. The interviewers did not have a medical background and were not medical professionals, which helped to limit power imbalances between researchers and participants. Written informed consent was obtained for all participants. The interview guide included questions about participant experiences with the health system; what ‘professionalism’ meant to them; and to share the domains of experience that were important or not important to them when considering professionalism in medicine. Interviews were audio recorded using digital voice recorders, transcribed using a secure transcription service ^25^ and field notes were taken during and after the interviews in reflexive field journals. Transcripts were anonymized and verified by the two research assistants who were both present for every interview. Transcripts were uploaded into NVivo 12 ^26^ for data management during the analysis phase. A thematic content approach to Qualitative Description was used as the method of analysis for the transcribed interview data ^27^. The analysis began by identifying small, descriptive codes which were then thematically synthesized to construct larger themes and sub-themes and confirmed through member checking and peer debriefing with all authors. Analysis was led by the first two authors and supervised by the senior author. Memos and annotations were also used throughout analysis to track progress and to identify researcher perspectives and thoughts on the data ^28^. Disconfirming cases were included in the generation and reporting of themes. The co-analysis ^29^ led to the production of new definitions of professionalism to be carried forward into the consensus-based process. There was an iterative incorporation of an increasing number of critical domains of professionalism identified from the interviews with research participants. Quotes reported in the results have been edited for clarity and readability. Data collection continued until saturation was reached, determined through iterative peer-debriefing on emerging themes throughout the data collection process.

### Phase Two Methods: Creating consensus on a new definition of professionalism

Collaborative consensus groups using a virtual modified nominal group technique (NGT) ^30, 31^ were held via secure web conferencing. NGT is a structured method to collect reliable information from experts in a focus group setting. By using NGT, researchers can elicit responses from each participant for predetermined, structured questions. Public members from equity-deserving groups were invited to take part, using the same recruitment strategies as in phase one of the study, in one of two consensus groups held in June and July 2022. Participants from phase 1 were invited to take part in the focus groups. Physicians and health systems stakeholders were also invited to participate in a third consensus group held in August 2022 through purposive sampling and advertisement via social media and the participating institutions’ equity, diversity and inclusion-related offices. The decision to facilitate community member and health provider groups separately was made to mediate power dynamics between community members and health care providers, and to maintain confidentiality.

At each consensus group meeting, the goal of updating the definition of medical professionalism to support health equity was presented to the entire group, along with a summary of the critical domains to be included in the definition resulting from the analysis of phase one data. Prioritization of domains occurred, followed by voting rounds. There was deliberation after each voting round to elaborate on benefits and unintentional consequences in situations where the voting was split, and further rounds of voting occurred until a unanimous consensus was reached.

The methods of this study align with O'Brien *et al.*, 2014 ^32^ standards for reporting qualitative research.

## Results

Phase one of the study included 28 participants. Interviews ranged from 16 minutes to 104 minutes in duration. Demographics for participants are presented in Table 1. The total of the self-identified groups exceeds the total number of interviews as some participants identified with multiple groups.

**Table 1.** Demographics: phase one interviews (n=28).

Category	
Age	
**Mean**	31.6
Gender	(n=27, one participant did not disclose gender)
Cisgender Man	5
Cisgender Woman	22
**Self-Identified Group**	
BIPOC	19
LGBTQ2S+	5
Disability/Health Condition	7
Newcomer/Refugee	2
Self-identified Religious Minority	2
**Location**	
British Columbia	1
Alberta	19
Ontario	7
Nova Scotia	1

BIPOC = Black, Indigenous, or Person of Colour; LGBTQ2S+ = Lesbian, Gay, Bi/Pansexual, Transgender, Queer, Two Spirit

### Phase One Findings: Understanding professionalism and health equity

Four main themes were generated from the interview analysis: health care at the margins; equity-oriented domains of professionalism; structural professionalism; and supporting improved professionalism.

Healthcare at the Margins

Throughout the interviews, participants discussed many instances of discrimination based on their identities. Participants spoke of how discrimination related to ability, culture and ethnicity, gender, poverty, intersectionality, geography, newcomer/immigrant status, race, and sexual orientation negatively impacted the delivery of services and caused them to feel excluded in clinical settings. Participants felt unwelcome because of assumptions made about them based on different aspects of their identity and observed similar dynamics among (non-physician) clinical staff.

“ *I don’t know if he was disturbed with stuff about his own staff, but he was like, he doesn’t like dealing with women of colour. That he associates them to, I dunno, ratchet cultures and the people that live in the ghetto*” (Participant 34, Female, BIPOC, 24 y/o)
“ *[The provider said]* ‘ *You don’t speak the language; we cannot help you’ and just [didn’t] provide me any services. And I wasn’t provided any more information.*” (Participant 2, Female, Newcomer/BIPOC, 30 y/0)

Participants described the emotional impact of their marginalizing experiences with providers, including feeling disrespected, anxious, and uncomfortable. In response to these experiences, some participants learned to advocate for better care for themselves and family in an attempt to decrease the impact of harmful services. Other participants sought care less often, leaving their medical issues untreated rather than dealing with health care providers.

“ *And I'm gonna be in some major pain, so now I'm worried about trying to train my husband about how to advocate for me with a healthcare provider about [medical condition] care when I can't maybe speak on my own behalf”* (Participant 7, Disability, Female, 34 y/o)
“ *But if I don't feel attended well, definitely I'll not be happy, I'll not go back there and I'll not recommend someone else there, and I'll not even entrust anyone going there”* (Participant 13, BIPOC, Female, 34 y/o)

Equity-Oriented Domains of Professionalism

Participants described the need for professionals to take their concerns seriously, starting from a place of respect and belief of the patient, rather than blaming or shaming patients for their health needs and practices. Many individuals described the feeling of their providers making assumptions and expressed that they would prefer physicians approach patient care with humility, accountability, and willingness to learn.

“ *Kind of feel like what are you supposed to do if the person that is supposed to look out for me and help me is not taking you seriously, what am I supposed to do?”* (Participant 28, Male, BIPOC/Religious minority, 21 y/o)
“ *If health care is really about helping people, then we really do need to stop the shame and blame no matter how severe something looks. Because when you don't get the support, you're not believed, or you get band aid fixes that typically end up causing other health concerns”* (Participant 24, Female, BIPOC/Disability 37y/o)
“ *If they could address their humanity and tell us when they don't know. I respect a doctor so much more when they say, "I'm sorry. I don't know but I can help you try and find that answer."”* (Participant 10, Female, Disability, 37 y/o)

Alongside attitudes, participants appreciated providers that took specific actions that signaled safe, anti-oppressive practice. For example, advocating on behalf of their patients when needed was a specific action that providers could take to increase trust. To participants, communication was a vital aspect of professionalism. Participants noted that body language, active listening, and facilitating discussion with patients were essential in creating a good relationship with their provider. However, behaviour like using technical language, withholding medical information, or talking down to patients disrupted relationality and was perceived as less professional.

“ *You don’t realize your rights as an individual in society. And so she [doctor] educated me on that, right? And then she provided me options from which to try. And we spent a year trying different things and its really improved my quality of life”* (Participant 27, Female, BIPOC/Disability, 43 y/o)
“ *We can’t say that each hospital will have a transgender nurse, transgender clinical doctor, it will become a little bit difficult. It lies on the existing doctors to like adjust and adapt to various people, various gender identity”* (Participant 25, Female, LGBTQ2S+, 24 y/o)
“ *Leaving a space like my family doctor as a kid, I think she was always talking. So [that is] hard to get a word in. Whereas just my family doctor now, she’ll slow down and leaves spaces”* (Participant 1, Gender not disclosed, Disability, 33 y/o)
“ *I feel like if I bring up something that's like minor and like I just wanna ask a question or whatever, it's like I feel like the response is like gonna be like, "Okay, this again. [Participant name]’s just crazy." But that's also (laughs) a me problem.”* (Participant 32, Female, LGBTQ2S+, 29 y/o)

Structural Professionalism

Participants identified many areas of medical culture that contributed to discrimination, including burnout, competition, protection of fellow colleagues, and patriarchal institutional culture. Participants emphasized the need for systemic resource allocation reforms that would focus on holding the system—rather than only the individual physicians—responsible.

“ *Taking care of yourself as a healthcare professional. So knowing when maybe you need a break when you’re not at your best, when you need someone to step in, or just making sure you are not coming to work because then you can take it up out on your patient”* (Participant 31, Female, BIPOC/Health Care Provider (HCP), 37 y/o)
“ *We need to have structures within our own, accountable organizations, whether they’re the provincial medical organizations or the national medical organizations, that are also strategically aligned with provinces and budgets to address all these widening concerns”* (Participant, 26, Female, BIPOC/HCP, 40 y/o)

Participants noted issues within the medical system that limited providers’ ability to act in professional ways, such as lack of diverse staffing and under-resourcing of clinical care. Participants discussed the need for effective accountability measures to prevent patient and staff concerns from being dismissed or deflected.

“ *We need some diverse humans in like who are not only like medical students or residents or whatever but also like nurses, LPNs, people at the front desk, reception people. Because they'll bring a totally different lived in experience.”* (Participant 7, Female, Disability, 34 y/o)
“ *I said "Well, I don't think it's appropriate that we generalize patients like that." And I said, "I don't think that's an accurate statement." And then, um, my, one of my colleagues was really upset that they had made that comment and actually wrote a complaint and then nothing, I don't think anything ever came of it. And same thing with another attending who made the [racist] comment to me, I filed a complaint with the university and nothing ever happened”* (Participant 14, Female, BIPOC/HCP, 23 y/o)

Supporting Improved Professionalism

Participants noted several ways to improve professional practice, including training and education. This included embedding perspectives of equity-deserving groups throughout undergraduate, graduate, and continuing medical education so providers are continually updated on professional practices.

“ *If we embedded these principles in the education from ground zero when these… students go and get into their science degrees, if they heard from the beginning, “patients matter, you're not always gonna agree with them”* (Participant 33, Disability, Female, 42 y/o)
“ *If universities could hold up to their statements that they’re making right now and make sure that those stay in place and infused not just in one course. Not just in one type of training session for one day. This is ongoing. This is a lifelong undoing”* (Participant 24, Female, BIPOC/Disability, 37 y/o)

### Phase Two Findings: Creating consensus on a new definition of professionalism

The identified domains and analysis generated from the interviews led to the creation of new definitions for medical professionalism which were presented to the focus groups. After each consensus group, the next iteration of the definition was presented to the following group, made up of different individuals. The demographics for the consensus groups are presented in Table 2. The total of the self-identified groups exceeds the total number of interviews as some participants identified with multiple groups.

**Table 2.** Demographics: phase two consensus groups (n=17).

Category	
Age (n=16, one participant age unknown)	
Mean	41.68
Gender	(n)
Cisgender Man	4
Cisgender Woman	12
Non-Binary/Gender Diverse	1
**Self-Identified Group**	
BIPOC	6
LGBTQ2S+	2
Disability/Health Condition	9
Self-identified Religious Minority	3
**Location**	
Alberta	13
Ontario	2
Nova Scotia	1
Quebec	1

Each meeting was approximately two hours in duration to allow for rich discussion with the goal of reaching consensus amongst the group members. Each domain identified in the interviews and described above was discussed with the group as to whether that domain was appropriately represented in the definition. These meetings resulted in the revised definition shown in Table 3, presented alongside the original definition of professionalism as defined by CanMEDS ^3, 4^.

**Table 3.** Original and revised definitions of professionalism.

Original Definition	As Professionals, physicians are committed to the health and well-being of individual patients and society through ethical practice, high personal standards of behaviour, accountability to the profession and society, physician-led regulation, and maintenance of personal health.
Revised Definition	As Professionals, physicians are dedicated to the holistic and equitable health and well-being of patients, colleagues, families, and communities. Physicians are obligated to remain learners to the discipline by challenging and improving their understanding of social and structural determinants of health and provide high-quality, strengths-based, and trauma-informed care. Professionalism is achieved through ethical collaboration with all partners in the achievement of health, with an explicit commitment to anti-racist and anti-oppressive practice. Physicians are dedicated to promoting diverse and psychologically safe clinical, learning, and research environments. Physicians hold accountability to patients, each other, and society, requiring a commitment to the integration of physicians’ personal health and professional demands. This accountability is actioned through valuing relationships and diversity, and through structural and systemic change that eliminates harm to patients and colleagues and creates opportunities for individuals and communities to thrive.

## Discussion

The results of this study indicate a misalignment between domains of professionalism needed by patients and those prioritized by physicians in the historical CanMEDS definition. Framing professionalism as a mechanism operating at a systems-level, in addition to the individual level, allows for a novel approach to eliminating the harm experienced by equity-deserving patients. Large-scale systemic action is urgently needed to create accountability structures to improve equitable, safe, and anti-racist environments throughout health systems, including medical education. For example, clinical teaching cases typically represent white, cisgendered, able-bodied patients and stereotype Black, Indigenous, and patients of colour, and provide insufficient training in cultural safety ^33, 34^. Clinicians’ behaviours, whether active (culturally and otherwise inappropriate or racist practices) or passive (lack of recognition or acknowledgment of structural barriers) contribute to inequity and damage relationships with equity-deserving groups in health care systems ^35, 36^.

The definition of professionalism informs personal, collegial, and societal expectations for physicians. As a result of this work, the authors advocate for incorporating anti-oppression and anti-racism as required areas of competence into the definition of professionalism in the CanMEDS framework. Physicians can then begin the necessary structural change that can substantively address inequities in healthcare and clinical outcomes for equity-deserving patients. The discrimination that the participants spoke about throughout this study similarly impacts medical learners, and as such redefining professionalism in the context of CBME addresses inequities in the training and professional experiences of equity-deserving physicians and learners ^37^. Enhanced expectations of professionalism throughout the medical education and practice trajectory enhances safety, allowing for greater representation in safer learning, practice and teaching spaces. Representation benefits patients with evidence of improved outcomes with patient-physician racial concordance ^38, 39^.

In addition to promoting optimal health and health experiences for all equity-deserving patients, the revised definition aligns with the Truth and Reconciliation Commission’s Calls to Action numbers 23 and 24, which call for increased and expanded education on Indigenous health, health inequities, and anti-racism ^40^, and the calls to justice from the National Inquiry on Missing and Murdered Indigenous Women and Girls, which recommend anti-racism and anti-bias training be incorporated into medical education ^41^. Moreover, Black medical learners at the University of Calgary have also made a call for anti-racist education and the Black Medical Students Association (BMSA) at the Cumming School of Medicine published Calls to Action ^42^ that explicate steps to create a more equitable and just profession; this has been reinforced by ongoing calls and advocacy at national levels ^37, 43– 45^. The commitment to equity in our new definition of professionalism would create an explicit expectation not only for anti-oppression and anti-racism education during undergraduate and postgraduate medical training, but also a need to maintain these competencies in throughout one’s medical career.

To achieve the operationalization of a new definition of professionalism, new educational content and pedagogy will need to be implemented to ensure medical students and residents achieve competency in anti-racism ^46^. Theoretical and skills-based anti-oppression teaching and assessment will be required at the level of the health care system at which the actions are directed: the micro- (e.g. patient-specific); meso- (e.g. community or population specific); and macro- (e.g. system) levels ^47^ within the CBME paradigm. By redefining professionalism to ensure competence in anti-oppression, which includes anti-racism, trainees and educators can be advocates through *agency* (“working the system”) and *activism* (“changing the system”) ^48^. As we understand oppression to work both on an individual and a systems level to create the most harm, shifting our lens of professionalism to include individual responsibility and the concept of structural professionalism, we argue this can create the most benefit to equity-deserving patients.

The strengths of the research team are numerous. The research team was composed of equity-deserving individuals, including Indigenous, Black and Persons of Colour to strive for research that was led, completed, and informed in such a way that the sensitivities and cultural safety of equity-deserving communities was prioritized. The deep community-engaged nature of this work provided a rigorous examination of public perceptions of physician professionalism. Several factors contribute to the rigor of this work ^22^. Rich data collection ensured thick description, enhancing the credibility and confirmability of the work. Frequent peer debriefings and the opportunity to embed member checking throughout the study reinforced the credibility and transferability of the work. The diverse participant representation further enhances the transferability of the data between medical schools. A limitation of this work is that it is a collaboration between only three of the 17 medical schools in Canada. This was mitigated by recruiting diverse participants through local networks and through national public advertisements. However, recruitment through social media and community partners could have excluded participants who were not engaged through those platforms, potentially impacting the demographics such as a low median age in our sample. The team acknowledges that further work should include a national consensus building process with all Canadian medical schools and development of aligned competencies and associated Entrustable Professional Activities (EPAs) and assessment. Important considerations include the use of artificial intelligence in designing and implementing assessments, appropriately engaging equity-deserving groups as assessors and reflective journaling to assess longitudinal learning. Further future implications of this work include a need for re-evaluation of regulatory and accountability frameworks. The new definition presented here de-emphasizes collegial accountability from the perspective of patients, so further engagement to understand how physicians support one another to actualize this vision of professionalism is required.

The results of this research advance medical education by contributing to the groundwork of an innovative adaptation of the *Professional* role, which has implications for professionalism as a competency in the CBME paradigm. Redefining professionalism not only advances equity among physicians but has implications for other health system-related areas of focus in Canada. This includes faculty hiring for medical educators within medical schools and influences broader areas such as health workforce planning by providing a benchmark of what professionalism competencies and accountability must look like. The community-based definition framed at a systems level that is presented here is a first step to achieve socially just medical education.

## Ethics and consent

This project was approved by the University of Calgary Conjoined Health Research Ethics Board (REB21-0036).Written informed consent was obtained for all participants.

## Data Availability

De-identified interview data will not be shared or accessible. While directly identifying details have been removed from the transcripts, access to the full transcripts is still a risk to confidentiality because of the subjects and contexts discussed. Participants consented to have their data used in the study analysis and reporting, but not for full transcripts to be shared outside the study team.
