# Supplementary material for: Redefining professionalism to improve health equity in competency based medical education (CBME): A qualitative study
Source: MedEdPublish (2016). 2024 Oct 18;14:237. [Version 1] doi: 10.12688/mep.20489.1 (PMC11589420; doi:10.12688/mep.20489.1)
Supplement: Peer review history

# Reviewer report: Reviewer response for version 1

**DOI:** 10.21956/mep.21928.r39497

**Published:** 2024-11-25

**Authors:** Bobbi Conner

**Affiliations:** 1 VA-MD College of Veterinary Medicine Virginia Tech, Blacksburg, Virginia, USA

The authors have submitted a well-written and extensive article that addresses an area of great need in healthcare education. I think the methods for identifying participants based on need (equity-deserving) rather than focusing on having a sample that is somehow proportionate to the relevant populations was wise. While not all groups can ever be represented, concerns about majority stakeholders’ perspective not being heard is not founded. For example, although there were comparatively few men in both groups, this should not be seen as a limitation per se. One could list many different categories that were not explicitly represented here, yet this doesn’t mean the final result is necessarily not representative of those voices. Indeed, the definitions of professionalism were developed to add to previously created definitions, such that earlier voices are clearly represented within.

 The authors have done a commendable job of describing their methods, making reproduction readily possible.

 The authors acknowledge several limitations and make minor recommendations for further work and assessment. I would argue some of the biggest limitations of the work include some vague phrases for what is meant in the final definition. More actionable verbs and assessable traits would be welcomed. For example, how would one assess whether a physician is “challenging and improving their understanding of social and structural determinants of health”? Similarly, how might one demonstrate that they are “promoting diverse and psychologically safe environments? These are challenging issues to address and more explicit descriptions – which admittedly may be available in other sources – would help with holding physicians accountable to such a definition.

 Overall, this is important work and the authors should be commended for their contribution.

Have any limitations of the research been acknowledged?

Yes

Is the work clearly and accurately presented and does it cite the current literature?

Yes

If applicable, is the statistical analysis and its interpretation appropriate?

Not applicable

Are all the source data underlying the results available to ensure full reproducibility?

No source data required

Is the study design appropriate and is the work technically sound?

Yes

Are the conclusions drawn adequately supported by the results?

Partly

Are sufficient details of methods and analysis provided to allow replication by others?

Yes

Reviewer Expertise:

NA

I confirm that I have read this submission and believe that I have an appropriate level of expertise to confirm that it is of an acceptable scientific standard.

# Reviewer report: Reviewer response for version 1

**DOI:** 10.21956/mep.21928.r39865

**Published:** 2024-11-07

**Authors:** Vijay Rajput

**Affiliations:** 1 Medical Education, Nova Southeastern University Dr Kiran C Patel College of Allopathic Medicine, Fort Lauderdale, Florida, USA

This is interesting study narrowly focused on CanMed competencies and making a case for redefining professionalism within that context. The word professionalism has struggle on global platform. The most definition overlap in the elements, traits or attributes and often lack consensus about their traits and elements based on social and cultural context. 

 Many of the current challenges of racism, oppression is related to universal social justice issue and also connected to explicit and implicit biases in individual behaviors irrespective of professional practice. 

 Addressing bias remains a fundamental professional responsibility of those responsible for the health of populations. Current interventions include instruction on the existence and harmful role of bias in perpetuating health disparities, as well as skills training for the management of bias.

 I believe that we don't need new definition of professionalism as we have hard time to define professionalism across the globe for all health professionals. The structural, political, social determinants of health cannot be fixed by changing definition of professionalism.

 The meaning and definition of professionalism is not static but dynamic and will continue to evolve as society will evolve. We physicians need to have servant leadership and practice within our sociocultural contextual professionalism as agreed within your society.  

 I will add this simple meaning of doctoring here: 

- “To be a whole man/woman who fulfills his/her task as scientist with professional quality and integrity; as human being, with kind heart, and high ideals; and as a member of society, with honesty and efficiency.”
  Felix Marti – Ibanez, New York 1960 (Courtesy Dr, Stuart Gordon)

Have any limitations of the research been acknowledged?

No

Is the work clearly and accurately presented and does it cite the current literature?

Yes

If applicable, is the statistical analysis and its interpretation appropriate?

I cannot comment. A qualified statistician is required.

Are all the source data underlying the results available to ensure full reproducibility?

Partly

Is the study design appropriate and is the work technically sound?

Yes

Are the conclusions drawn adequately supported by the results?

Partly

Are sufficient details of methods and analysis provided to allow replication by others?

Yes

Reviewer Expertise:

Medical education, Humanism,  and bedside medicine

I confirm that I have read this submission and believe that I have an appropriate level of expertise to confirm that it is of an acceptable scientific standard, however I have significant reservations, as outlined above.

## References

[1] : Eliminating Explicit and Implicit Biases in Health Care: Evidence and Research Needs. *Annu Rev Public Health* .2022;43: 10.1146/annurev-publhealth-052620-103528 477-501 10.1146/annurev-publhealth-052620-103528 35020445 PMC9172268

# Reviewer report: Reviewer response for version 1

**DOI:** 10.21956/mep.21928.r39498

**Published:** 2024-11-02

**Authors:** Giesela Grumbach

**Affiliations:** 1 Governors State University, University Park, Illinois, USA

This article offers timely insights into equity in medical professionalism. To address the limitations, it would have been good to have more men represented in this study. While more male representation would have addressed some limitations, it remains well-constructed, well-written, and well-designed for reproducibility. 

 To improve male representation for future studies, consider targeting recruitment through the use of diverse milieus.

Have any limitations of the research been acknowledged?

Partly

Is the work clearly and accurately presented and does it cite the current literature?

Yes

If applicable, is the statistical analysis and its interpretation appropriate?

Not applicable

Are all the source data underlying the results available to ensure full reproducibility?

Yes

Is the study design appropriate and is the work technically sound?

Yes

Are the conclusions drawn adequately supported by the results?

Yes

Are sufficient details of methods and analysis provided to allow replication by others?

Yes

Reviewer Expertise:

Racial and cultural equity and qualitative methods.

I confirm that I have read this submission and believe that I have an appropriate level of expertise to confirm that it is of an acceptable scientific standard.
